# Comparison of quantitative and qualitative scoring approaches for radiation-induced pulmonary fibrosis as applied to a preliminary investigation into the efficacy of mesenchymal stem cell delivery methods in a rat model

**DOI:** 10.1259/bjro.20210006

**Published:** 2021-07-05

**Authors:** Li Ming Wang, Sungmi Jung, Monica Serban, Avishek Chatterjee, Sangkyu Lee, Krishinima Jeyaseelan, Issam El Naqa, Jan Seuntjens, Norma Ybarra

**Affiliations:** 1 Research Institute of the McGill University Healthcare Centre, Montréal, Canada; 2 Department of Pathology, McGill University Healthcare Centre, Montréal, Canada; 3 Medical Physics Unit, Cedars Cancer Centre, McGill University Healthcare Centre, Montréal, Canada; 4 Memorial Sloan Kettering Cancer Centre, New York, NY, USA; 5 Radiation Oncology, University of Michigan – Ann Arbor, Ann Arbor, MI, USA; 6 Medical Physics Unit, Cedars Cancer Centre, Montréal University Healthcare Centre, Montreal, Canada; 7 Research Institute of the McGill University Healthcare Centre & Medical Physics Unit, CedarsCancer Centre, McGill University Healthcare Centre, Montreal, Canada

## Abstract

**Objectives:**

Compare a quantitative, algorithm-driven, and qualitative, pathologist-driven, scoring of radiation-induced pulmonary fibrosis (RIPF). And using these scoring models to derive preliminary comparisons on the effects of different mesenchymal stem cell (MSC) administration modalities in reducing RIPF.

**Methods:**

25 rats were randomized into 5 groups: non-irradiated control (CG), irradiated control (CR), intraperitoneally administered granulocyte-macrophage colony stimulating factor or GM-CSF (Drug), intravascularly administered MSC (IV), and intratracheally administered MSC (IT). All groups, except CG, received an 18 Gy conformal dose to the right lung. Drug, IV and IT groups were treated immediately after irradiation. After 24 weeks of observation, rats were euthanized, their lungs excised, fixed and stained with Masson’s Trichrome. Samples were anonymized and RIPF was scored qualitatively by a certified pathologist and quantitatively using ImageScope. An analysis of association was conducted, and two binary classifiers trained to validate the integrity of both qualitative and quantitative scoring. Differences between the treatment groups, as assessed by the pathologist score, were then tested by variance component analysis and mixed models for differences in RIPF outcomes.

**Results:**

There is agreement between qualitative and quantitative scoring for RIPF grades from 4 to 7. Both classifiers performed similarly on the testing set (AUC = 0.923) indicating accordance between the qualitative and quantitative scoring. For comparisons between MSC infusion modalities, the Drug group had better outcomes (mean pathologist scoring of 3.96), correlating with significantly better RIPF outcomes than IV [lower by 0.97, *p* = 0.047, 95% CI = (0.013, 1.918)] and resulting in an improvement over CR [lower by 0.93, *p* = 0.037, 95% CI = (0.062, 1.800].

**Conclusion:**

Quantitative image analysis may help in the assessment of therapeutic interventions for RIPF and can serve as a scoring surrogate in differentiating between severe and mild cases of RIPF. Preliminary data demonstrate that the use of GM-CSF was best correlated with lower RIPF severity.

**Advances in knowledge:**

Quantitative image analysis can be a viable supplemental system of quality control and triaging in situations where pathologist work hours or resources are limited. The use of different MSC administration methods can result in different degrees of MSC efficacy and study outcomes.

## Introduction

For patients undergoing thoracic radiotherapy (RT), radiation-induced pulmonary fibrosis (RIPF) is an important, permanent, and late lung toxicity. RIPF is characterized by increased collagen deposition and the loss of pulmonary functionality following tissue remodeling. The prolonged and complex processes which leads to RIPF has made it a challenge to assess and treat.

RIPF is traditionally and still currently assessed through two main modalities: (1) *in vivo* imaging, such as CT or MRI and (2) *in vitro* visualization, such as collagen staining via a histopathology technique such as Masson’s Trichrome. Both modalities require subjective appraisal by trained specialist, such as a radiologist or pathologist, in order to derive discrete outcomes on RIPF severity and extent. This poses a problem for studies utilizing RIPF as an objective outcome. Not only is the required involvement of a specialist difficult, due to limited resources and time, but there can be variations in the outcomes of subjective grading.^1,2^ As such, methods of RIPF scoring and assessment should be critically investigated.

In regard to the treatment of RIPF, in recent years, infusions of mesenchymal stem cells (MSCs) have shown to have therapeutic value in treating fibrotic diseases.^3–6^ The administration of MSCs and their localization to sites of injury were reported to be of benefit to injured organs^7–9^ including the lungs.^10,11^ In lung-specific fibrosing diseases, MSCs have demonstrable efficacy in reducing bleomycin-induced pulmonary fibrosis when administered via the tail vein^12,13^ and intratracheally.^14^ In the case of RIPF, two main mechanisms of MSC action are of interest: (1) replacement of pneumocytes lost due to cell death following injury and 2) MSC immunomodulatory properties.

In regards to the first mechanism, MSCs, being multipotent cells, have potential to differentiate into a variety of cell types^15^ giving them the ability to regenerate, through engraftment, differentiation and replacement of, local cell populations.^12^ It has been reported that 15% of MSCs administered will differentiate into type-II pneumocytes and replenish lost pneumocytes, in an *in vivo* mouse model utilizing 2 × 10^5^ MSCs intravenously injected via the tail vein soon after irradiation.^16^ While retention does not correlate with functional improvement,^17^ it is possible that temporarily replacing cell populations local to regions of damage may exert a positive effect locally.^12^

In regards to the second mechanism, MSCs home to sites of injury due to local release of chemokines^18^ and, from there, MSCs release soluble anti-inflammatory factors and exhibit immunomodulatory properties at the site of injury.^3,6,19^ The immunomodulatory effects, through increased expression of anti-inflammatory cytokines and through paracrine effects have been reported to be of therapeutic benefit in acute kidney,^18^ liver,^20^ and lung^5,21^ injury animal models and animals models of sepsis,^22^ chemical damage^23^ and physical damage.^24,25^

However, much of the studies regarding MSC effects focus on administration, whether intravenous or intratracheal, of allogeneic MSCs. There is also the interesting alternative of using granulocyte-macrophage colony stimulating factor (GM-CSF) to induce the mobilization of endogenous MSCs to sites of injury. GM-CSF exerts its effects as a cell signaling molecule. GM-CSF’s presence leads to stimulation of immune cells, such as alveolar macrophages,^26^ and recruitment of multipotent stem cells, such as MSCs, capable of restoring the alveolar components^27^ local to the region of injury. GM-CSF have been reported to reduce the severity of lung injury,^28^ chemical and hemorrhagic acute lung injury^29^ and the severity of bacterial lung infection.^30^ In addition, deficiencies in GM-CSF have been linked to more severe fibrosis outcomes in bleomycin-induced pulmonary fibrosis models.^31,32^

Despite studies reporting techniques for RIPF scoring, there are minimal studies conducted to compare different scoring techniques. Within this paper, we will attempt to validate the effectiveness of a well-reported quantitative method of blue collagen quantification, as stained by Masson’s Trichrome, and compare it to the qualitative RIPF scoring of a certified pathologist. After which we will apply both the quantitative and qualitative scoring techniques to derive a preliminary comparison of different MSC infusion methods and their association with RIPF and collagen deposition outcomes. For this part, we will compare two different routes of MSC administration (intravascular and intratracheal) and an endogenous MSC recruitment method (through the use of GM-CSF) to evaluate if they correlate to changes in collagen deposition, a hallmark of RIPF severity.

## Methods and materials

### MSC preparation

The Animal Care and Use Committee of the University of McGill approved the animal protocol. 8-week-old Sprague-Dawley (Charles River Laboratories, QC, CA) male rats (*n* = 4) were first anesthetized by isoflurane, then euthanized by CO_2_ asphyxiation. The femur and tibia marrow cavities of the rats were exposed under sterile conditions and flushed using a 10 ml syringe attached to 20G needle containing MSC Growth medium (MSCGM^™^ Lonza, Cedarlane, CA) with antibiotics/antimycotic (Invitrogen, ON, CA). The fluid was collected and passed through a 70 µm cell strainer. The cells were washed thrice by centrifugation at 400g for 5 min, using MSCGM medium containing antibiotics/antimycotics. The cells were cultured in T-75 culture flasks at 37°C, 5% CO_2_ in MSCGM plus antibiotics/antimycotics. The medium was changed for the first time after 48 h to eliminate non-adherent cells, and twice a week subsequently. The cells were trypsinized and subcultured at 80–90% of confluence. Only cells from passage 2 were used to achieve a balance between cellular homogeneity and size.

### MSCs characterization

MSC were characterized for osteogenic, adipogenic and chondrogenic potential following the methodology of a previous study.^33^ The specific method of preparation is detailed in Supplementary Material 1.

### Animal preparation

25 pathogen-free female Sprague-Dawley rats, aged 7–8 weeks, weighing 200–300 g, were housed in the institution’s animal facility. Animals were fed food and water *ad libitum*. After an acclimation period of 1 week, animals were randomly assigned into 5 experimental groups with 5 rats per group (*n* = 5): control group given no irradiation (CG); control group given radiation without any treatment intervention (CR); one group treated with intraperitoneal GM-CSF (drug); one group given intravascularly administration MSCs (IV); and one group given intratracheally administrated MSCs (IT).

**Figure 1.**
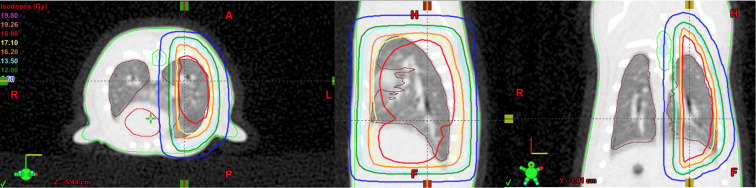
Animal treatment plan in the transverse (left), sagittal (middle) and coronal plane (right). Image includes dose distributions, with isodose line values indicated in the image (left), as well as contours for the lungs, spinal cord, spinal cord PRV and liver. PRV, planning risk volume.

**Figure 2.**
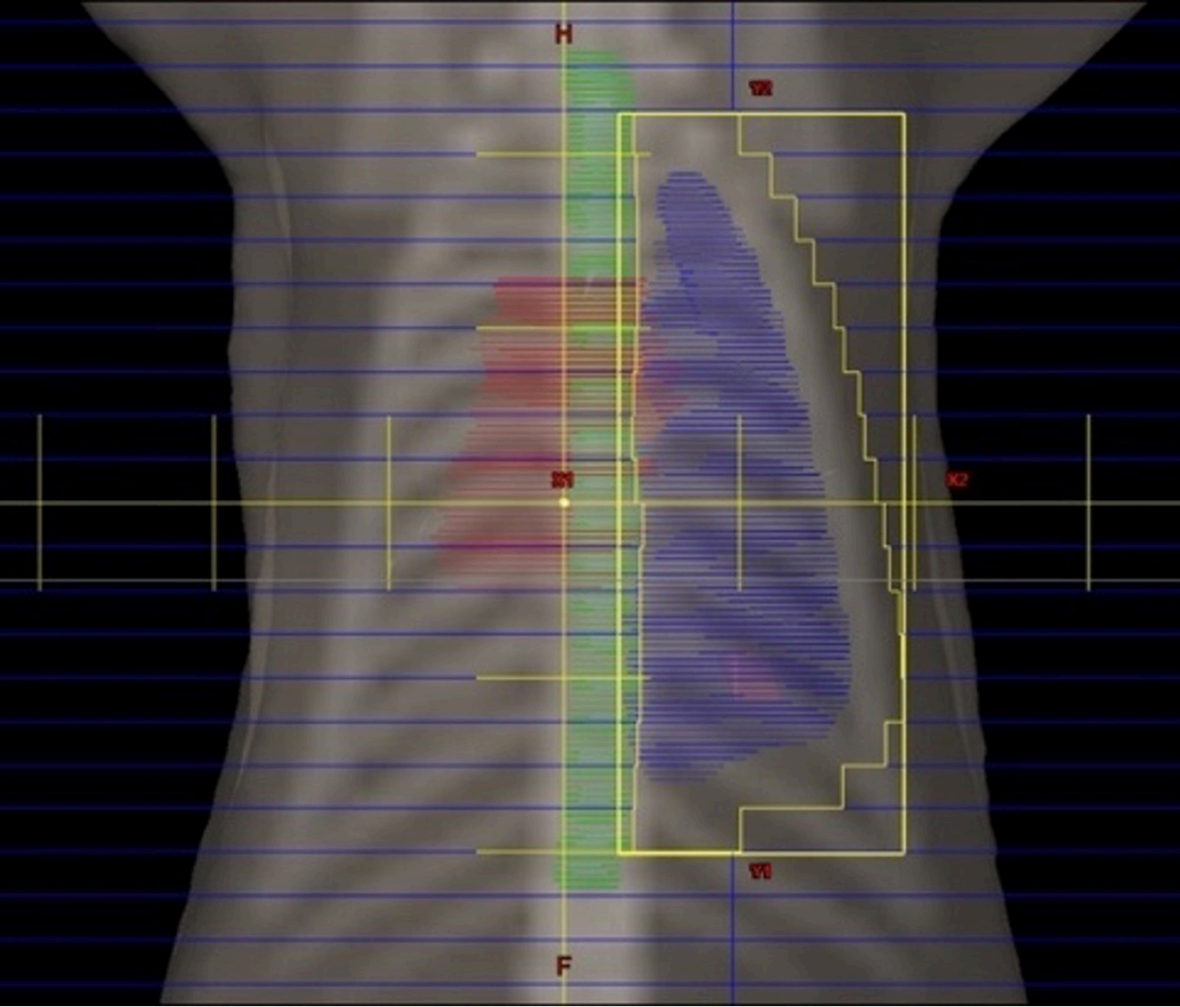
Beams eye view featuring the outline of the treated lung (blue), spinal cord PRV (green) and heart (red) as well as the multi leaf collimator and leaf positions (identified by the lateral yellow outline and the blue bars, respectively). PRV, planning risk volume.

1 week before irradiation, baseline CT scans were taken for radiation treatment planning. For this purpose, all groups except CG were induced to anesthesia with isoflurane. Once anesthetized, the animals were imaged on a Philips Brilliance Big Bore CT simulation scanner (Philips Medical Systems, Bothell, Washington, DC) following an optimized small animal protocol (120 kVp X-ray tube voltage, 175 mA tube current, 0.37 mm in-plane resolution, 0.8 mm axial resolution). The animals were placed in a prone position on an in-house built Styrofoam holder with reference markers for positioning reproducibility. The lungs, heart and spinal cord were contoured on the baseline CT images. An example of a treatment plan is shown in Figure 1. A single fraction of 18 Gy was prescribed to the right lung using a 6 MV photon beam (Novalis Tx linear accelerator). 18 Gy was chosen as it was shown in our own pilot studies and literature^34–36^ to induce consistent pulmonary fibrosis suitable for experimentation. A hemi-thorax parallel-opposed 3D conformal treatment plan was designed (Eclipse^TM^ V 11.0) for each individual animal based on the CT image. Each plan was adapted to the individual animal’s anatomy to spare the spinal cord, heart and left lung. An example of the beam’s eye view is shown in Figure 2. The prescribed dose was delivered using the Novalis Tx linear accelerator (Varian Medical Systems, Palo Alto, CA). Anesthetized rats were positioned relative to the markers established during the planning CT. For each rat prior to irradiation, final positioning accuracy was established using cone beam CT.

Immediately after irradiation, the rats received the following treatment intervention: the Drug group received an initial intraperitoneal dose of 10 µg/kg of GM-CSF followed by a daily administration of the same dose for a total of 7 days.^37^ The IT and IV groups received 2 × 10^5^ and 1 × 10^6^ cells respectively, immediately after irradiation and once every week after irradiation for 6 weeks. Infusion duration was established via pilot studies to ensure complete infusion of MSC dose while ensuring that the animals remain healthy. The number of cells injected was based on previous studies using intravenous administration,^8,13,38^ intratracheal administration^38–40^ and pilot studies which determined the safe number of cells to be injected. Follow-up lung CT imaging was taken every 2 weeks for a total of 24 weeks.

### Histological preparation

After 24 weeks, rats were euthanized by CO_2_ asphyxiation after anesthetization with isoflurane. The chest cavity was opened, and the lungs were excised, washed in PBS, transversally segmented, fixed in 10% paraformaldehyde, and paraffin embedded. Lungs were segmented into upper, middle and lower sections. While the entire lung was sliced and mounted, only six sections, or two slides, from each of the upper, middle and lower sections were obtained. An additional seven slides were also stained to replace poorly mounted slides that had damaged samples. In total, this accounted for 157 samples.

For preparation of histological analysis, lung section slides were deparaffinized, rehydrated through a graded alcohol series, and stained with Masson’s Trichrome following the manufacturer’s protocol.^41^ Staining of all 157 samples occurred over 7 sessions, 20–25 slides per session, where samples were stained following manufacturer protocol using reagents that were not reused more than twice to ensure comparability between slides. Slides were then dehydrated through a graded alcohol series, cleared in xylene and mounted.

### Pathologist scoring

A certified pathologist scored RIPF for all 157 stained lung sections using the modified Ashcroft Scale for presence and severity of pulmonary fibrosis.^2^ Samples were anonymized prior to scoring. Given the heterogenous and patchy presence of collagen deposition characteristic of RIPF, the region with the most severe RIPF characteristics is scored a grade from 0 to 8 using the modified Ashcroft Scale described in Table 1. Pathologist scoring was performed using a 20-fold objective optimized for histological assessment of lung fibrosis.^2^

**Table 1.** A table of the modified Ashcroft scale directly adopted from Hübner et al^2^ with criteria used by the pathologist to determining RIPF scoring

RIPF grade	Descriptions of grade
0	Alveolar septa: no fibrotic burden at the flimsiest small fibers in some alveolar walls Lung structure: normal lung
1	Alveolar septa: isolated gentle fibrotic changes (septum <3x thicker than normal) Lung structure: alveoli partly enlarged and rarified, but no fibrotic masses
2	Alveolar septa: clear fibrotic changes (septum >3x thicker than normal) with knot-like formation but not connected to each other Lung structure: alveoli partly enlarged and rarified, but no fibrotic masses
3	Alveolar septa: contiguous fibrotic walls (septum >3x thicker than normal) predominantly in whole microscopic field Lung structure: alveoli partly enlarged and rarified, but no fibrotic masses
4	Alveolar septa: variable Lung structure: single fibrotic masses (≤10% of microscopic field)
5	Alveolar septa: variable Lung structure: confluent fibrotic masses (>10% to≤50% of microscopic field). Lung structure severely damaged but still preserved
6	Alveolar septa: variable, mostly non-existent Lung structure: large contiguous fibrotic masses (>50% of microscopic field). Lung architecture mostly not preserved
7	Alveolar septa: non-existent Lung structure: alveoli nearly obliterated with fibrous masses but still up to five air bubbles
8	Alveolar septa: non-existent Lung structure: microscopic field with complete obliteration with fibrotic masses

RIPF, radiation-induced pulmonary fibrosis.

The scale focuses on appraisal of the alveolar septa and overall lung structure.

### Software analysis

**Figure 3.**
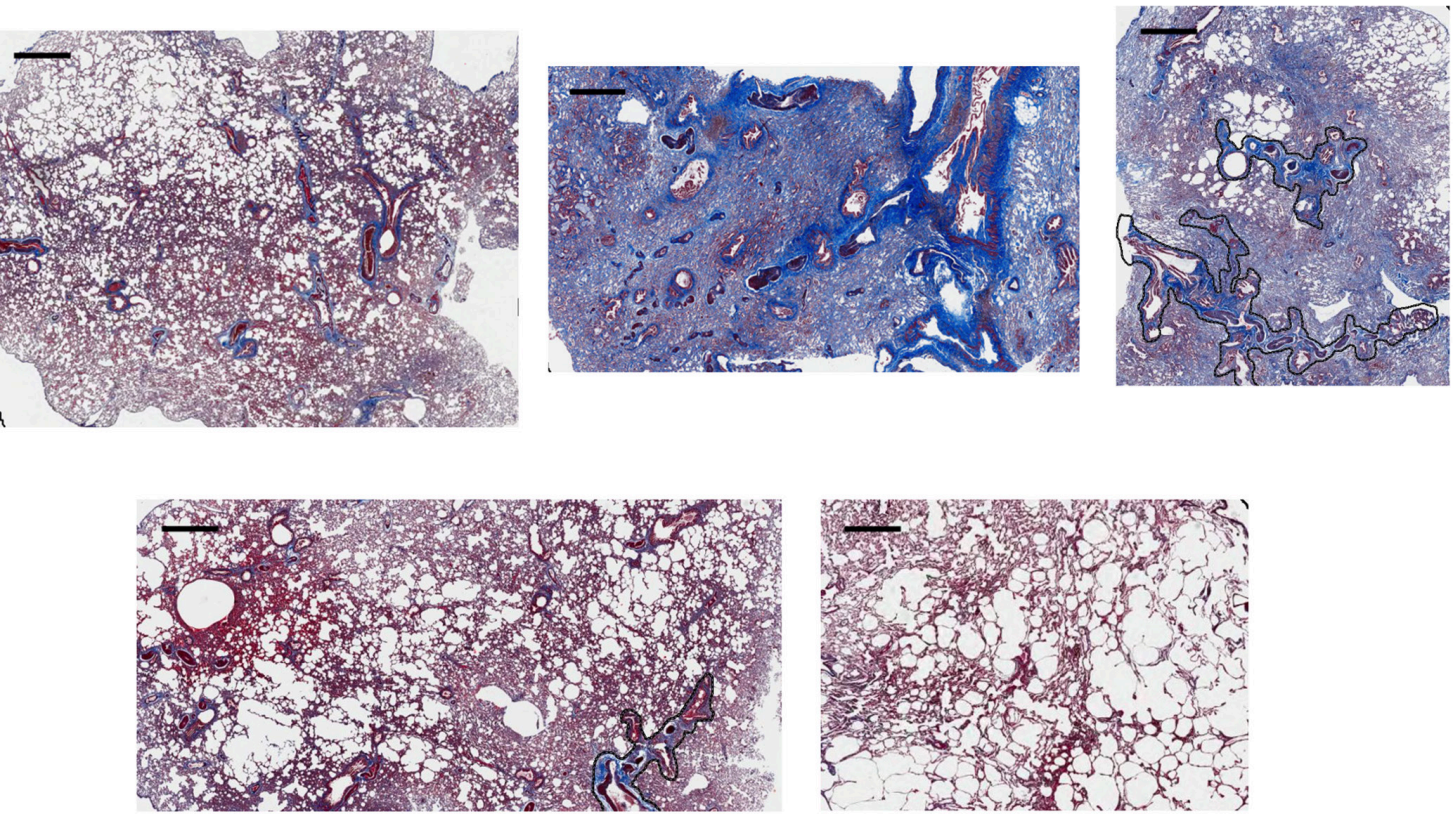
Images of Masson’s Trichrome stained lung samples from each group: CG (top left), CR (top middle), DRUG (top right), IV (bottom left) and IT (bottom right). Regions delineated by the black lines within the alveolar fields are identified vessel groups containing arterioles, venioles and bronchioles which are excluded from analysis. The black scale bars in the upper left corner of all the images are representative of 250 µm.

**Figure 4.**
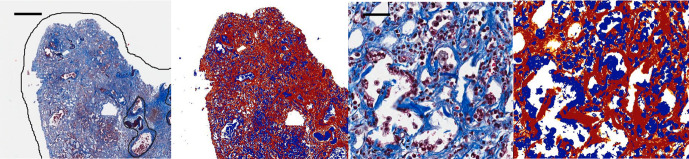
Images depicting the threshold analysis procedure performed by the software. The first alveolar image (first from the left) is analyzed by the software (visualized in the second image from the left) via a system of color thresholding where strong positives (red), moderate positives (orange) and weak positives (yellow) are detected, according to how closely it resembles our set HSB parameter. The strength of the positive region is associated with a coefficient that is applied to the total area of that strength of positive before it is totaled and used to derive the ratio value of P_R_. The same technique is shown in a magnified field in the two images on the right. Regions delineated by the black lines within the alveolar fields, of the leftmost image, are identified vessel groups containing arterioles, venioles and bronchioles which are excluded from analysis. The black scale bars, in the top left of the leftmost and second from the right images, are 250 µm and 50 µm respectively for the left two images and the right two images. HSB, hue, saturation and brightness.

Images of the same 157 prepared samples were digitally captured at 20× magnification (Figure 3) using a whole slide scanning technique (Aperio^TM^ Leica Biosystems, Buffalo Grove, IL, USA). The captured images were then imported to and analyzed for collagen as stained by aniline blue using ImageScope (Leica Biosystems, Buffalo Grove, IL, USA) with predetermined parameters for specific hue, saturation and brightness specifically for detection of aniline blue (please see Supplementary Material 1. for specific parameters). The parameters values were established empirically through visual verification and allowed for the exclusion of the alveolar lumen from analysis. Prior to quantitative analysis, histological images were contoured to remove all major vessels and airways roughly greater than 250 µm in diameter, leaving only the alveolar regions with small vessels and airways for analysis (Figure 4). This analysis generated a P_R_ value per sample that was considered our quantitative scoring (exact derivation of P_R_ is provided in the Supplementary Material 1).

### Statistical analysis

The qualitative scores provided by the certified pathologist, and the quantitatively assessed P_R_ values calculated by the ImageScope software, with predetermined parameters, were assigned to the five treatment groups. Data were analyzed using Stata/IC (v. 15.1, College Station, TX) statistical software with the exception of the binary classifiers which were completed using an in-house developed MATLAB code (R2018a, MathWorks, Massachusetts). The association between pathologist scoring and algorithm scoring (P_R_) is assessed by limits of agreement (95% prediction intervals) using a linear regression model. To further validate if the P_R_ scores were indeed grading RIPF phenomena using visual cues that were similar to that used by the pathologist, Naïve Bayes and Fit Discriminator binary classifiers were trained, using P_R_ value to predict pathologist scoring binned into two categories: Mild (including grades of 0 to 4 on the modified Ashcroft) and severe (including grades of 5–8). Ratios of mild to severe cases were kept consistent between training and testing sets with two thirds of the data set randomly appointed to be used as the training cohort and the rest as testing. Mean differences between the treatment groups were assessed using variance components analysis and mixed models (with treatment group as systematic effect and rat as random effect). Group scoring means and 95% confidence interval of the mean were calculated. A *p*-value < 0.05 was considered significant.

## Results

The MSCs differentiated into osteoblasts, adipocytes, and chondrocytes, confirming that the cells used in this study were indeed MSCs (please see Supplementary Material 1 for more details).

### Qualitative and quantitative scoring agreement

**Figure 5.**
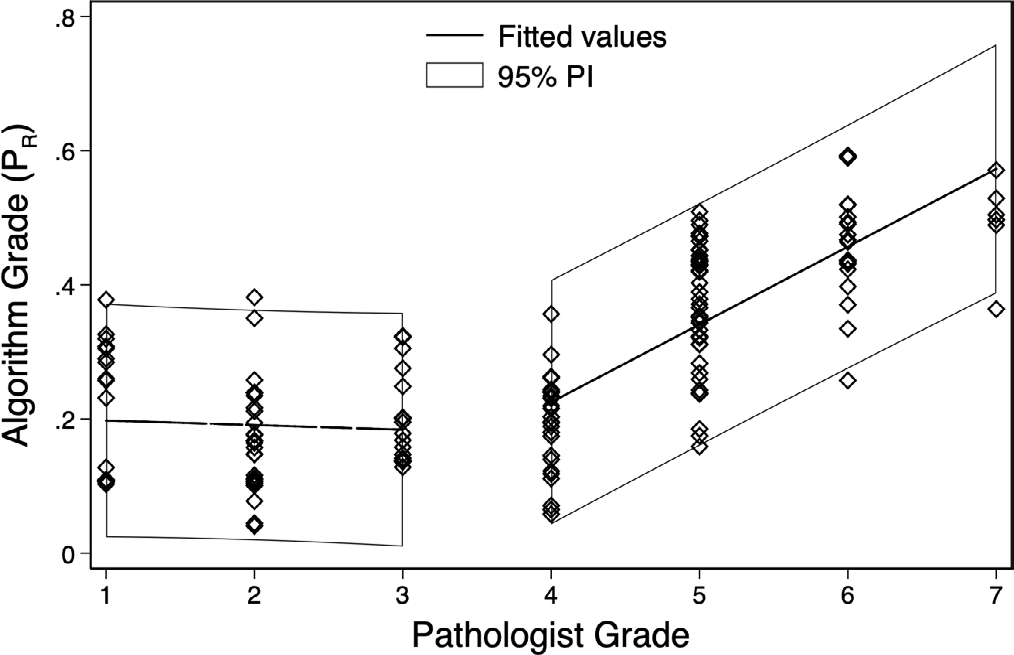
Plot of the association between pathologist scoring, via the modified Ashcroft Scale, and P_R_ values for all graded patients. Each point represents an assessed sample with the x-axis value indicating the pathologist score and the y-axis value indicating the P_R_ value. The best fitting linear regression line and the 95% PI are displayed. PI, prediction interval.

Pathologist grading using the modified Ashcroft scale, was associated with the P_R_. Pathologist scoring and P_R_ were plotted with a fitted line and 95% predictive intervals (Figure 5). P_R_ for a pathologist score of 4 was 0.225 ± 0.177 (95% prediction interval), with a 0.116 (*p* < 0.0001) increase per unit increase in the pathologist scoring. There appears to be agreement between pathologist scoring and P_R_ for pathologist grades from 4 to 7. Lower pathologist graded samples do not have agreement.

### Classifier performance

**Figure 6.**
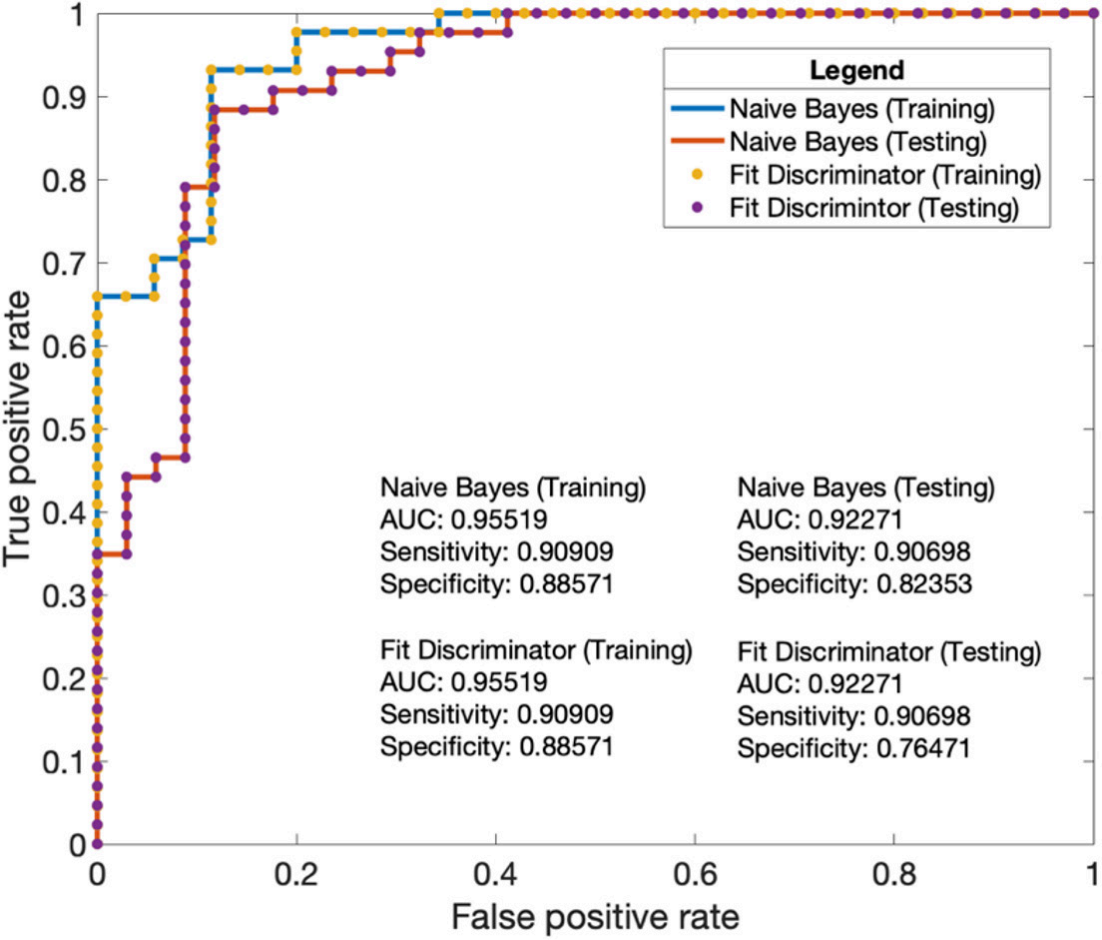
Analysis of the binary classification ability for P_R_ scoring. The two classifiers performed similarly on both the training and testing set, with the naïve Bayes classifier (blue line and red line) performing similarly. With the naïve Bayes (red line) performing slightly better than the fit discriminator (purple dot) on the testing set.

The Naïve Bayes model performed slightly better than the fit discriminator (Figure 6) in terms of specificity. Overall, both the naïve Bayes and fit discriminator performed similarly in terms of area under the receiver operating characteristic curve (AUC) outcomes and sensitivity. Analysis through binary classification of P_R_ scoring indicated that, on the testing set, both the classifiers achieved a high AUC of 0.92.

### Comparison of administration modality on RIPF outcomes

**Figure 7.**
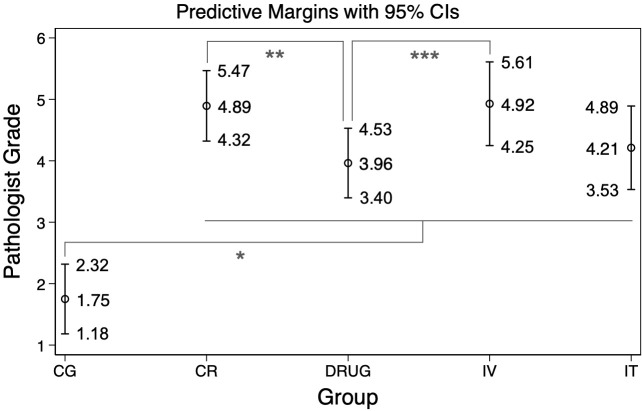
Mean of the modified Ashcroft Scale scores for each treatment group, presented with a 95% CI for the mean value, as assessed by a certified pathologist. Significant differences between groups (as indicated in Table 2) are signified by the asterix. CI, confidence interval.

The Drug group achieved the lowest score while being significantly different from both CG, CR and IV (*p*-value << 0.05). IT achieved low scores, comparable to that of Drug, but were not significantly different from either CR, Drug or IV (*p*-value of 0.151, 0.589, 0.162 respective). While IV had the most severe RIPF outcomes, being very similar to the CR group in terms of scores and being only significantly different from the Drug group (*p*-value < 0.05). Figure 7, Table 2 provide a detailed summary of the pathologist’s scoring results.

**Table 2.** Pathologist scoring mean differences between treatment groups with 95% CIs and corresponding *p* values estimated by the mixed model. Statistically significant differences are indicated in bold

	Pathologist scoring mean difference	95% CI	*p*-value
**CR *vs* CG**	**3.14**	**(2.274, 4.014)**	**0.000**
**DRUG *vs* CG**	**2.21**	**(1.347, 3.079)**	**0.000**
**IV *vs* CG**	**3.18**	**(2.225, 4.132)**	**0.000**
**IT *vs* CG**	**2.46**	**(1.511, 3.414)**	**0.000**
**DRUG *vs* CR**	**−0.93**	**(−1.800,–0.062)**	**0.037**
IV *vs* CR	0.03	(−0.921, 0.991)	0.940
IT *vs* CR	−0.68	(−1.636, 0.272)	0.151
**IV *vs* DRUG**	**0.97**	**(0.013, 1.918)**	**0.047**
IT *vs* DRG	0.25	(−0.701, 1.199)	0.589
IT *vs* IV	−0.72	(−1.747, 0.315)	0.162

CI, confidence interval.

## Discussion

In this study, we validated the utility of a reported^42,43^ quantitative assessment technique that uses total area of fibrotic regions, as stained by Masson’s trichrome, and compared it with a gold-standard assessment completed by a certified pathologist using the modified Ashcroft scale. While grading derived from the quantitative method was able to distinguish between cases of severe, defined as an Ashcroft grade >= 4, and non-severe fibrosis, with a grade <4, it was not as capable of discerning between closely related RIPF severities. This is because the color-driven quantitative analysis is insensitive to changes of the interstitial structures while a pathologist can appraise changes to interstitial structures to further specify scoring. An example of this is the assessment of the alveolar septa thickness and the assessment of overall alveoli structure organization in scoring RIPF.^2^ These structural characteristics cannot be appraised by quantitative analysis unless these structural changes result in an aggregation of collagen and aniline blue stain which only occurs when alveoli structures are affected by severe RIPF. Thus, rendering mild RIPF events with minimal, but still qualitatively perceptible structure changes or damage to be not appraised during quantitative scoring. Quantitative scoring methods are also susceptible to inconsistencies in tissue processing, staining intensities and variations in sample fixation as these variances cannot be easily accounted for when establishing parameters for analysis. However, despite the quantitative scoring method being not sensitive enough to match the pathologist’s scoring performance, the technique does have two main advantages over traditional pathologist scoring: (1) it is automatable and (2) it is able to assess the condition of entire samples, as opposed to specific areas limited by the viewfinder. These properties can, and in our study did, make for a great supplemental system of quality control and triaging in situations where large volumes of samples require more than the available pathologist work hours or resources. Overall, our use of this method within this study does further validate the utility of a quantitative RIPF assessment.

Using our validated, we then conducted a novel comparison of MSC administration methods and their relationship to RIPF outcomes. The modes of administration we compared were intravenous injection, intratracheal administration and endogenous recruitment by use of GM-CSF. We found that GM-CSF was correlated with significantly reduced collagen deposition and RIPF severity.

Foremost, it was notable to find that intravenous injection of MSCs appears to result in outcomes no different than our non-treated irradiated controls. We suspect that this is due to the high rates of MSC entrapment within the lungs leading to adverse events. During our pilot studies, we observed animal deaths due to lung embolisms after intravascular injections (data not shown). Our observations corroborate reports of MSC entrapment^44^ that have led to vascular obstruction,^45^ formation of microthrombi,^46^ pulmonary embolisms and death.^25,47^ Currently, the thrombogenic activity of intravenous MSC infusion are not well understood^48^ and, as such, the potential impact on RIPF outcomes is also not well understood. However, despite the literature and our observations, recent pre-clinical studies validating MSC’s therapeutic effects predominantly use intravenous infusions^49–51^ as method of MSC delivery. As such, the use of intravenous infusion of MSCs as a method in pre-clinical mice models investigating stem cell therapeutic potential should be revisited. And, future studies should focus on determining optimal formulations that maximize therapeutic effects while minimizing deleterious outcomes.

Intratracheal administration of MSCs appears to be associated with better outcomes than the intravenous technique. We suspect that strong retention of the MSCs in the lung following intratracheal administration^25^ in combination with circumvention of the vascular and thrombogenic side-effects could be a plausible explanation for improved outcomes with the intratracheal method. As such, future mechanistic studies featuring more time points should be conducted to clearly identify homing of intratracheally administered MSCs and if homing indicates retention in specific parts of the lung and if these regions of retention differ from intravascular or GM-CSF modalities.

The modality associated with the best outcomes has been observed to be GM-CSF. In the case of our study, GM-CSF’s benefits may be due to the circumvention of limitations related to the intravenous and intratracheal modalities. In comparison with the intravenous modality, the greatest advantage with GM-CSF is that there are no issues of thrombogenic interactions in the vasculature. In comparison with the intratracheal technique, GM-CSF utilizes a simpler procedure of intraperitoneal injections and avoids risk of mechanical damage to the trachea due to the need for intubation. While this does appear promising, there is report that GM-CSF can worsen RIPF due to increased immune response in sites of injury.^52^ As such, more studies are needed to investigate the mechanistic effects of GM-CSFs as it pertains to RIPF. Specifically, the fluctuations of GM-CSF levels throughout injury, inflammation and tissue repair processes associated with RIPF.

There are limitations to our preliminary study comparing MSC infusion modalities. Foremost, we did not have a defined equivalent dosage level at which we expect to see similar RIPF or collagen deposition outcomes across the three delivery modalities. As such, we did not pursue any mechanistic data such as MSC homing, inflammatory cell infiltration, quantity of entrapped or retained cells or data regarding cytokine levels. These limitations are inherent to our study which is intended to be a novel comparison of infusion modalities and how they may correlate to RIPF outcomes as assessed through blinded pathologist assessment and objective quantitative measure of collagen deposition. There are currently no studies, to our knowledge, comparing these modalities of treatment and as such, there have been no standard metric to suggest what a similar dose of MSC would be in relation to these modalities or whether a dose delivered through one modality would create a comparable cytokine or immune response, which ultimately leads to comparable RIPF outcomes. As such, our study is an initial offering to motivate future comparative work, providing a point of reference for future studies which seek to more accurately and better compare these treatment modalities.

## Conclusion

Quantitative image analysis is beneficial to the assessment of therapeutic interventions for RIPF and can serve as a scoring surrogate in differentiating between severe and mild cases of RIPF. In addition, preliminary results indicate that GM-CSF is correlated with the least severe RIPF outcomes and may be the most effective MSC delivery modality for RIPF in comparison to two other method of MSC delivery. Intravenous administration of MSCs does not appear to be effective at reducing RIPF severity.
